# Corrosion Behavior of Typical Engineering Structural Steels in a Plateau Valley Atmospheric Environment

**DOI:** 10.3390/ma19061142

**Published:** 2026-03-15

**Authors:** Xiayan Wang, Xuexu Xu, Lili Zhang, Junjie Cai, Bingkun Yang, Hongchi Ma, Cuiwei Du, Zhiyong Liu, Xiaogang Li

**Affiliations:** 1 National Materials Corrosion and Protection Scientific Data Center, Key Laboratory for Corrosion and Protection (MOE), University of Science and Technology Beijing, Beijing 100083, China; 2 School of Materials Science and Engineering, China University of Petroleum (East China), Qingdao 266071, China; 3 China Electric Power Research Institute, Beijing 100192, China

**Keywords:** structural steel, plateau valley atmospheric environment, uniform corrosion, stress corrosion, corrosion mechanism

## Abstract

This study systematically investigated the corrosion behavior of three typical engineering structural steels (Q235, Q420, and Q420qENH) in the plateau valley atmospheric environment of the Sichuan–Tibet region using field exposure tests (including uniform corrosion and stress corrosion coupon tests), electrochemical measurements, and microscopic characterization. The results reveal that the three steels underwent predominantly uniform corrosion, accompanied by pitting corrosion, regardless of the presence of stress. Compared with Q235 and Q420 steels, Q420qENH exhibits superior corrosion resistance, which is ascribed to the denser rust layer formed as a result of its corrosion-resistant composition. Under tensile stress, both the uniform corrosion rate and pit dimensions increased significantly relative to stress-free conditions, demonstrating a pronounced accelerating effect of stress on uniform and localized corrosion. Furthermore, the enrichment of sulfur within the rust layer at the pit bottoms suggests the involvement of atmospheric SO_2_ in the localized corrosion process, which aggravates the breakdown of the rust layer and the acidification of the local environment at the pit bottom, thereby promoting pit growth.

## 1. Introduction

Sichuan–Tibet regions and the southeastern regions of the Qinghai–Tibet Plateau are core strategic zones for major national projects, including the Sichuan–Tibet Railway and Yalong River hydropower development. These areas are characterized by high-altitude valley environments with elevated humidity, frequent condensation, and poor dispersion of atmospheric pollutants, which collectively create aggressive conditions for metallic corrosion. In addition, environmental and operational factors such as wind, thermal stress, and traffic loading expose steel structures to combined degradation modes including uniform corrosion, localized corrosion, and stress corrosion cracking (SCC). Understanding the atmospheric corrosion behavior of structural steels in such plateau valley environments is therefore essential for ensuring the long-term durability and safety of critical infrastructure.

Atmospheric corrosion of structural steels generally occurs under thin, inhomogeneous electrolyte layers and is governed by factors such as temperature, relative humidity, chloride deposition, and pollutant concentrations [1,2,3,4]. Under these conditions, the rapid diffusion of oxygen can promote SCC initiation at corrosion pit bottoms, leading to higher SCC susceptibility compared with immersion environments [5]. Previous studies have shown that elevated temperature and relative humidity accelerate the corrosion of structural steels [6,7,8]. Increased temperature leads to the formation of a more porous rust layer, while higher relative humidity promotes the rapid transport of corrosive species to the steel surface; both contribute to an increased corrosion rate. Chloride deposition has been identified as a key factor in reducing corrosion resistance [9,10,11], and under cyclic wet–dry conditions, chloride accumulation can increase the akaganéite (β-FeOOH) content in rust layers, degrading their protectiveness [12,13]. In polluted atmospheres, SO_2_ infiltration can further create pathways for oxygen and aggressive ions (e.g., O_2_, Cl^−^) to reach the steel substrate, thereby accelerating corrosion [14,15]. These studies collectively demonstrate that environmental corrosives often alter rust layer composition or structure, facilitating access of corrosive media to the steel surface and influencing the corrosion rate.

In addition to environmental factors, steel composition plays a critical role in atmospheric corrosion resistance [16,17]. Alloying elements such as Ni and Al have been shown to improve corrosion performance in saline atmospheres [18,19], primarily by modifying the rust layer’s compactness and phase composition. For instance, Ni additions promote the formation and aggregation of NiFe_2_O_4_, which facilitates the transformation of γ-FeOOH into more protective α-FeOOH [19]. This is further supported by the results of atmospheric exposure tests conducted by Yang et al. [20] on Q235 and Q420 steels. Similarly, Mo helps preserve protective phases such as NiFe_2_O_4_ in chloride-containing environments [21], and elements including Cu and Mo can densify the rust layer, enhancing its barrier effect against aggressive ions [5]. Despite these insights, research on the corrosion behavior and corrosion-resistant design of structural steels specifically in plateau valley environments remains limited [22]. Investigating the role of alloy design under these environmental conditions is therefore crucial for guiding material development in support of major engineering projects.

In this work, the corrosion behavior of typical engineering structural steels is systematically studied in a representative Sichuan–Tibet plateau valley environment through corrosion kinetics, morphological characterization, and corrosion product analysis. The effects of steel strength, corrosion-resistant alloy design, and applied tensile stress on corrosion performance are evaluated. This study was conducted in a representative plateau valley environment that shares key climatic and geographical features with similar regions worldwide. The findings therefore offer transferable insights into the corrosion behavior of structural steels, with significant implications for material selection and durability assessment in analogous environments globally.

## 2. Materials and Methods

### 2.1. Materials

Three commercially available structural steel plates were studied: plain carbon steel Q235, low-alloy structural steel Q420, and low-alloy weathering steel Q420qENH. The chemical compositions of these steels are listed in Table 1. Specimens with dimensions of 10 × 10 × 3 mm^3^, sectioned from each steel were ground using SiC abrasive paper up to 2000 grit, followed by mirror polishing with diamond suspensions of 2.5 μm and 1.5 μm. The polished surfaces were then etched with 4% nitric acid in ethanol, thoroughly rinsed with deionized water and ethanol, and air-dried. Metallographic examination was performed using an FEI Quanta 250 scanning electron microscope (SEM, FEI, Hillsboro, OR, USA).

### 2.2. Field Atmospheric Corrosion Testing

Baiyu County (Garzê Tibetan Autonomous Prefecture) is located in the transitional zone between the Qinghai–Tibet and Yunnan–Guizhou Plateaus, within the northern Hengduan Mountains, with the majority of the region lying at altitudes above 3500 m. The regional climate features an annual average temperature of 12.3 °C, a diurnal temperature range of 18–20 °C, a relative humidity of 57%, and an annual precipitation of about 600 mm, with clearly defined wet and dry seasons, according to historical records provided by the meteorological bureau. The Jinsha River Bridge (98.78° N, 31.25° E) in Baiyu County, a critical node in the Sichuan–Tibet power grid and transportation network, represents a typical large-scale engineering application of structural steel in a plateau valley environment. Based on these environmental and engineering characteristics, the bridge site was selected for the atmospheric corrosion exposure tests conducted in this study.

**Figure 1:**
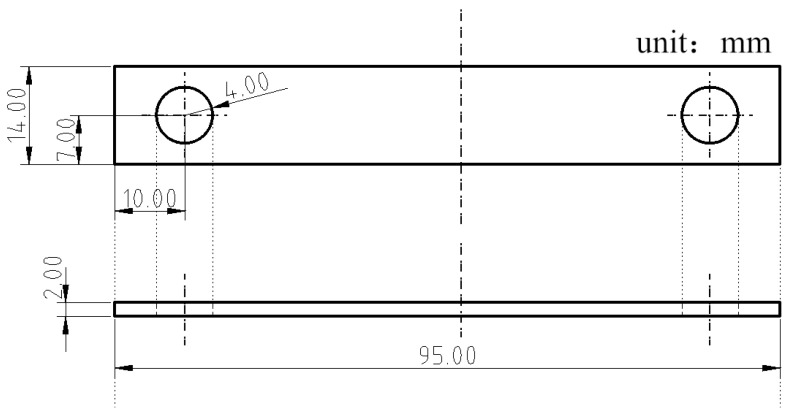
Schematic illustration of the U-bend specimen dimensions for stress corrosion testing.

**Figure 2:**
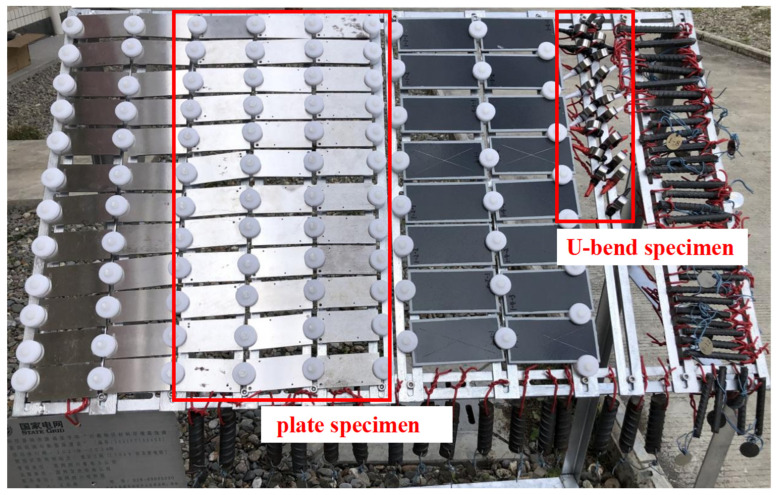
Photographs of field-exposed plate specimen and U-bend specimens of the three structural steels. The exposure started and ended in March, covering a full two-year period. All specimens were installed at an angle of 45° to the horizontal, facing south, following the requirements of ASTM G50.

To simulate stress-free and stressed service conditions, rectangular plate stress-free specimens (100 × 50 × 3 mm^3^) and U-bend specimens (dimensions shown in Figure 1) were fabricated from the three steels. For the plate specimens, four parallel samples were exposed per steel type and exposure period, while two parallel U-bend specimens were used for each test condition. Prior to field exposure, all specimens were ground to 2000-grit SiC paper, ultrasonically cleaned in deionized water and ethanol, and dried in air. The initial dimensions and mass of each plate specimen were measured using a vernier caliper and an analytical balance, respectively. All specimens were then installed at the outdoor test site, as shown in Figure 2, and exposed to the plateau valley atmosphere for periods of 1 and two years. The exposure started in March 2021 and ended in March 2023, covering a full two-year period. All specimens were installed at an angle of 45° to the horizontal, facing south, following the requirements of ASTM G50. After retrieval, the samples were subjected to corrosion-rate calculation, morphological characterization, corrosion-product analysis, and electrochemical tests to evaluate their corrosion behavior and mechanisms.

### 2.3. Corrosion Rate

The uniform atmospheric corrosion rates of the three steels were assessed using the weight-loss method. After field retrieval, the plate specimens were chemically descaled by immersion in a solution consisting of 500 mL concentrated HCl, 500 mL deionized water, and 3 g hexamethylenetetramine for 5 min to remove corrosion products, in accordance with ASTM G1-25 [23]. Following descaling, the specimens were re-weighed, and the corrosion rate *v* was calculated as follows:

(1) $$v=\frac{(w_{0}-w_{t})\times 8.76\times 10^{4}}{S\rho t}$$

where *w*_0_ represents the initial weight (g) of the plate specimen, *w_t_* is the weight (g) after corrosion product removal, *S* is the surface area (mm^2^) of the specimen, *ρ* is the material density (taken as 7.9 g/cm^3^), and *t* is the exposure time (years) in the atmosphere. Three parallel samples were used for weight loss measurements to calculate the average corrosion rate.

The U-bend specimens were sectioned along the central axis, and the exposed cross-sectional surface was sequentially ground to 2000-grit SiC paper and mirror-polished using diamond pastes (2.5 μm and 1.5 μm). The remaining thickness after corrosion was measured with an Olympus 400 confocal laser scanning microscope (CLSM, Olympus, Tokyo, Japan). Five random locations were measured on each specimen, and the average value was used for subsequent calculations. The corrosion rate *v* for the U-bend specimens was calculated according to the following formula:

(2) $$v=\frac{a_{0}-a_{t}}{t}$$

where *a*_0_ is the initial thickness (mm), *a_t_* is the average remaining thickness after exposure (mm), and *t* is the exposure time (years).

### 2.4. Corrosion Morphology Inspection and Corrosion Product Analysis

After atmospheric exposure, the surface and cross-sectional morphologies of the rust layers on both plate and U-bend specimens were examined by SEM. The chemical composition of the rust layers was analyzed by energy-dispersive X-ray spectroscopy (EDS) attached to the SEM, utilizing mapping analyses. Following the removal of corrosion products with the descaling solution described in Section 2.3, the morphology of the exposed corrosion pits was observed by SEM and further quantified using CLSM. For statistical evaluation, at least 60 pits were randomly selected from no fewer than five different fields of view, and the widths and depths were measured. Additionally, the maximum pit depth at the arc top of each U-bend specimen was determined.

The phase composition of the corrosion products was identified by X-ray diffraction (XRD) using a SmartLab diffractometer (SmartLab, Tokyo, Japan), with patterns collected over a 2θ range of 10–90° at a continuous scan rate of 5°/min.

### 2.5. Electrochemical Testing

Electrochemical measurements were carried out in a conventional three-electrode setup consisting of a platinum electrode (20 mm × 20 mm) as the counter electrode and a saturated calomel electrode (SCE) as the reference electrode. The working electrode was a 10 mm × 10 mm × 3 mm specimen prepared from each steel, with the exposed surface ground to a 2000-grit finish. Tests were performed in a simulated aqueous solution formulated according to the water quality of the Jinsha River [24] (main chemical constituents of the simulated solution are listed in Table 2). All tests, including open-circuit potential (OCP) monitoring, electrochemical impedance spectroscopy (EIS), and potentiodynamic polarization, were conducted sequentially using a CS electrochemical workstation (Corrtest, Wuhan, China). EIS spectra were recorded over a frequency range of 100 kHz to 10 mHz with a 10 mV sinusoidal perturbation. Potentiodynamic polarization curves were scanned from −1.2 V to −0.2 V (vs. SCE) at a rate of 0.5 mV/s.

## 3. Results

### 3.1. Microstructural Characterization

**Figure 3:**
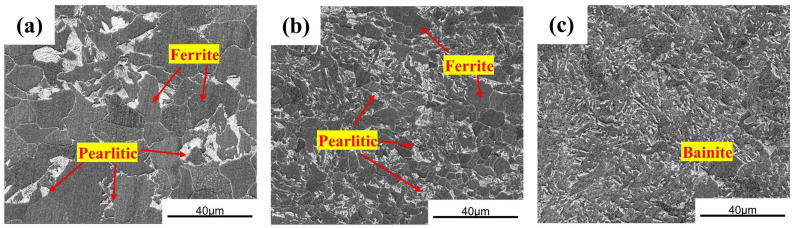
SEM images showing the microstructures of the three structural steels: (**a**) Q235, (**b**) Q420, and (**c**) Q420qENH.

Figure 3 displays the microstructures of the three structural steels. While both Q235 and Q420 steels exhibit a typical ferrite–pearlite microstructure (Figure 3a,b), the Q420qENH weathering steel comprises a predominantly bainitic structure (Figure 3c). Compared with Q235, the Q420 steel possesses a finer grain size and a more dispersed pearlite distribution. This refinement can be attributed to its lower carbon content, which inhibits the growth of lamellar pearlite [25]. In contrast, the addition of copper in Q420qENH promotes bainite formation [26], leading to its distinct microstructural characteristics.

### 3.2. Corrosion Rates

**Figure 4:**
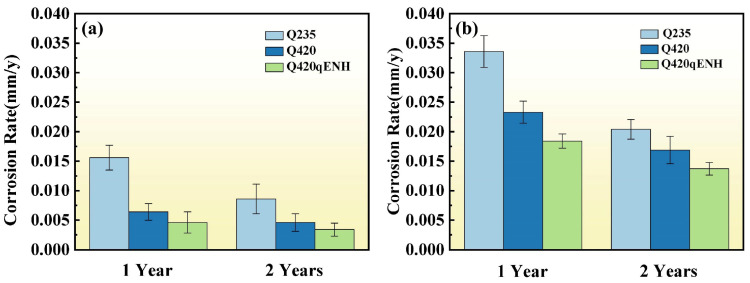
Corrosion rates of the three structural steels in the plateau valley atmosphere after one year and two years of exposure: (**a**) plate specimens and (**b**) U-bend specimens.

The corrosion rates of the three structural steels after one year and two years of exposure in the plateau valley atmospheric environment, calculated using Equations (1) and (2), are summarized in Figure 4. For Q235 steel, the first-year corrosion rate of 0.0156 mm/y corresponds to corrosivity category C2 (low) according to ISO 9223 [27], reflecting the relatively mild nature of the inland plateau valley atmosphere. A consistent corrosion resistance ranking is observed: Q235 < Q420 < Q420qENH. Following the second year of exposure, the corrosion rates decreased to 55.1%, 71.8%, and 56.6% of the respective first-year values for Q235, Q420, and Q420qENH, indicating the progressive development of a more protective rust layer over time. Notably, the corrosion rates of the U-bend specimens were significantly higher than those of the flat coupons, demonstrating that applied tensile stress markedly accelerates corrosion. This finding is consistent with previous research [28].

### 3.3. Corrosion Morphology

#### 3.3.1. Corrosion Morphology of Plate Specimens

**Figure 5:**
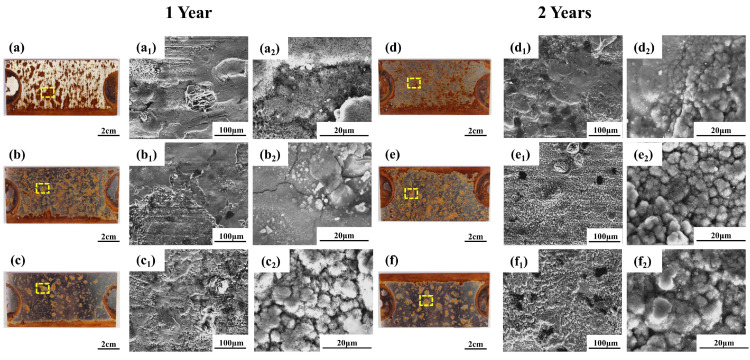
Macroscopic photographs and corresponding SEM micrographs of the rust layers on plate specimens after (**a**–**c**) 1-year and (**d**–**f)** 2-year exposures: (**a**,**d**) Q235, (**b**,**e**) Q420, (**c**,**f**) Q420qENH. Sub-figures with subscript ‘1’ and ‘2’ denote low- and high-magnification SEM images, respectively. Micrographs were taken from a representative localized corrosion area within the yellow-boxed region shown in the macroscopic image.

The macroscopic surface morphologies of the rust layers formed on the plate specimens of three structural steels after one- and two-year exposures are presented in Figure 5a–f. All steels exhibited widespread yellowish-brown rust spots. Compared with Q235 and Q420, which showed denser and more continuous rust coverage, Q420qENH displayed fewer and more isolated rust spots. Representative low- and high-magnification SEM images of the rust layers are provided in Figure 5a_1_–f_1_,a_2_–f_2_, respectively. Notably, Q420qENH developed a denser rust layer after only one year of exposure (Figure 5c_1_). With prolonged exposure to two years, the rust layers on all steels became progressively denser and more uniform (Figure 5a_1_–f_1_). At higher magnification (Figure 5a_2_–f_2_), the rust layers of all three steels reveal an interwoven, needle-like morphology. This characteristic structure is consistent with that of lepidocrocite (γ-FeOOH), as reported in the literature [29].

**Figure 6:**
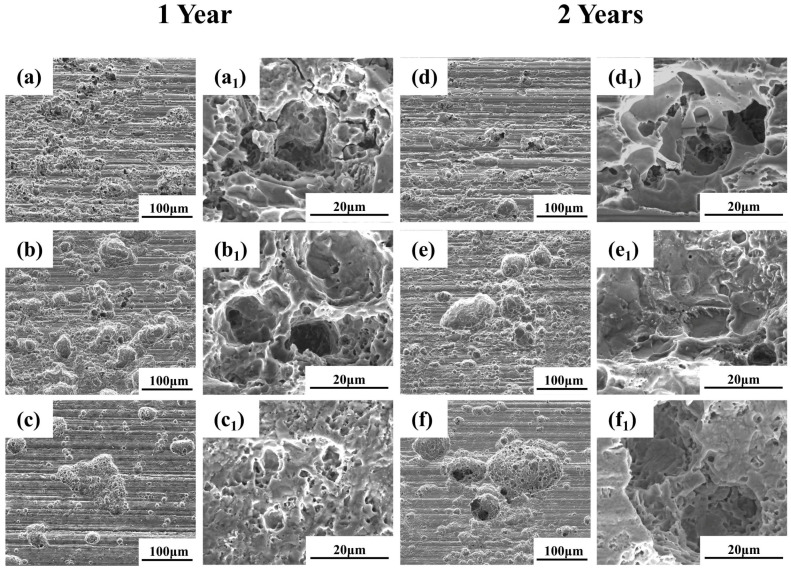
SEM images of the descaled surfaces of plate specimens after (**a**–**c**) 1-year and (**d**–**f**) 2-year exposures: (**a**,**d**) Q235, (**b**,**e**) Q420, (**c**,**f**) Q420qENH. Sub-figures with subscript ‘1’ denote higher magnification views of pit morphologies.

Corrosion pit morphology directly reflects the protective quality of the rust layer. Figure 6 presents SEM images of the descaled plate specimens, revealing the localized corrosion morphology after rust removal. After one year of exposure (Figure 6a–c), both Q235 and Q420 steels display densely distributed pits with relatively small diameter, though pits on Q420 are slightly larger. In contrast, the surface of Q420qENH steel comprises a few large-diameter pits along with numerous smaller ones. After two years of exposure (Figure 6d–f), the pit diameter on all steels increases. Moreover, smaller but deeper secondary pits form within larger primary pits, indicating that the overlying corrosion products provide limited protection to the substrate beneath the pits.

**Figure 7:**
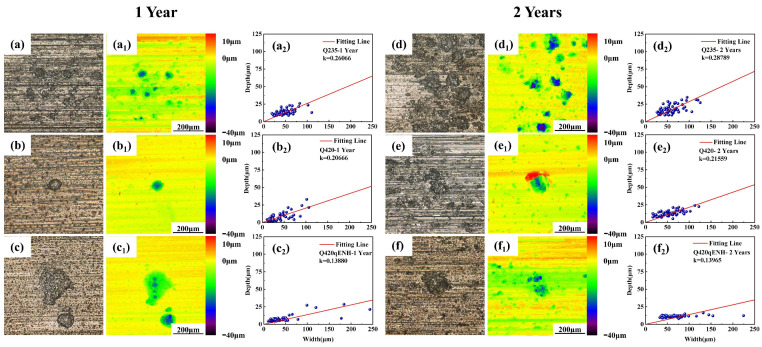
CLSM analysis of pits on descaled plate specimens: surface morphology (**a**–**f**), corresponding profile maps (**a_1_**–**f_1_**), and statistical distributions of pit depth vs. diameter (**a_2_**–**f_2_**). Results are shown for (**a**–**c**) 1-year and (**d**–**f**) 2-year exposures: (**a**,**d**) Q235, (**b**,**e**) Q420, (**c**,**f**) Q420qENH. The coefficient k represents the depth-to-diameter ratio.

For three-dimensional characterization and growth-kinetics analysis, the descaled plate specimens were examined by CLSM. The resulting surface morphologies and corresponding pit-size statistics are shown in Figure 7. The CLSM images (Figure 7a_1_–f_1_) reveal distinct pit characteristics: Q235 shows a high density of small diameter but relatively deep pits; Q420 has fewer, slightly deeper pits; and Q420qENH exhibits significantly larger-diameter, shallower pits.

Pits that grow preferentially in depth pose a greater threat to structural integrity [30]. Therefore, the depth-to-diameter ratio was analyzed as a measure of pitting susceptibility. A linear fit was applied to the depth–diameter data, with the slope (*k*) representing this ratio. The statistical results (Figure 7a_2_–f_2_) yield the following ranking for the depth-to-diameter ratio: Q235 > Q420 > Q420qENH, indicating that Q420qENH provides the best resistance to localized corrosion penetration in this environment. Prolonged exposure slightly increases the depth-to-diameter ratio for all steels, suggesting that pit growth becomes progressively more depth-oriented over time.

#### 3.3.2. Corrosion Morphology of U-Bend Specimens

**Figure 8:**
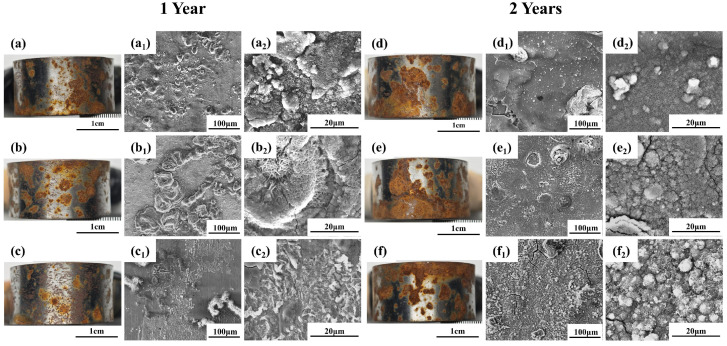
Macroscopic photographs and SEM micrographs of rust layers on the arc top regions of U-bend specimens after (**a**–**c**) 1-year and (**d**–**f**) 2-year exposures: (**a**,**d**) Q235, (**b**,**e**) Q420, (**c**,**f**) Q420qENH. Sub-figures with subscript ‘1’ and ‘2’ denote low- and high-magnification SEM images, respectively.

The macroscopic rust layers formed on the U-bend specimens after one- and two-year exposures are shown in Figure 8. After one year, reddish-brown linear and yellowish-brown patchy corrosion products developed on all specimens. Compared with Q235 and Q420, the Q420qENH specimen exhibited a higher proportion of reddish-brown linear products and fewer patchy deposits. By the second year, the linear reddish-brown products on all specimens had evolved into more extensive yellowish-brown patchy products.

Representative SEM images of the rust layers at different magnifications are presented in Figure 8a_1_–f_1_,a_2_–f_2_. With prolonged exposure, the rust morphology transitioned from a linear pattern to a denser, more layered structure. Notably, the rust layer on Q420qENH appeared denser, consistent with the trend observed for the corresponding plate specimens (cf. Figure 5). At higher magnification (Figure 8a_2_–f_2_), the corrosion products of all steels reveal an interwoven, needle-like morphology, suggesting that lepidocrocite (γ-FeOOH) is a primary constituent.

**Figure 9:**
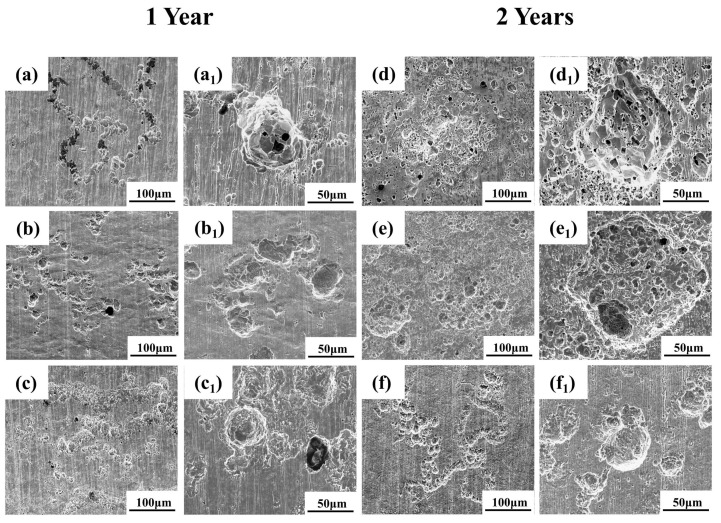
SEM images of the descaled arc top regions of U-bend specimens after (**a**–**c**) 1-year and (**d**–**f**) 2-year exposures: (**a**,**d**) Q235, (**b**,**e**) Q420, (**c**,**f**) Q420qENH. Sub-figures with subscript ‘1’ denote higher magnification views.

To examine localized corrosion under combined atmospheric and stress conditions, the arc top regions of the descaled U-bend specimens were imaged by SEM (Figure 9). Beneath the former linear corrosion products, the substrate showed a continuous distribution of fine pits. With prolonged exposure, these pits increased in number and size, gradually coalescing and completing the transition from a linear to a patchy distribution. Higher magnification views (Figure 9a_1_,d_1_,d_2_) reveal that secondary pits initiated within larger ones, and this phenomenon was also observed in plate specimens. This further suggests that the rust layer provides limited protection to the underlying substrate.

**Figure 10:**
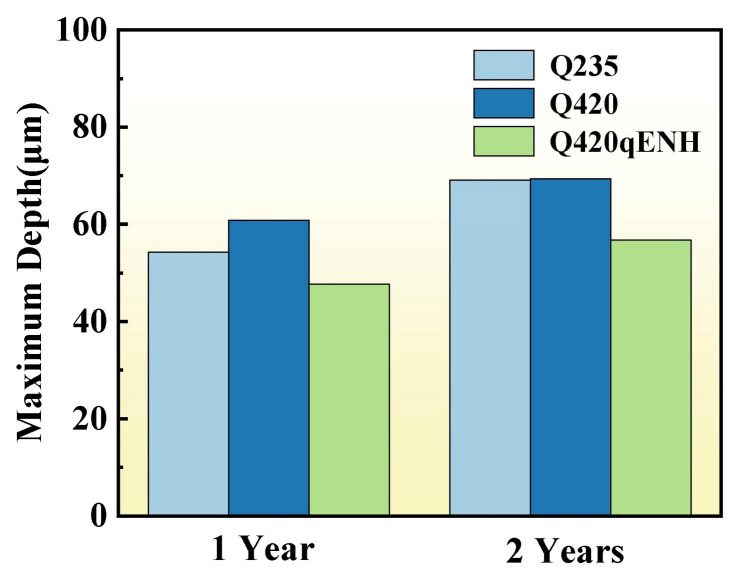
Maximum pit depths measured at the arc top regions of U-bend specimens after one year and two years of exposures.

To evaluate pitting corrosion behavior under stress, the maximum pit depths at the arc top of the U-bend specimens were measured; the results are summarized in Figure 10. The maximum pit depth follows the order: Q420 > Q235 > Q420qENH, indicating that under applied stress, pits in Q420 steel exhibit the strongest propensity for depth-wise propagation.

**Figure 11:**
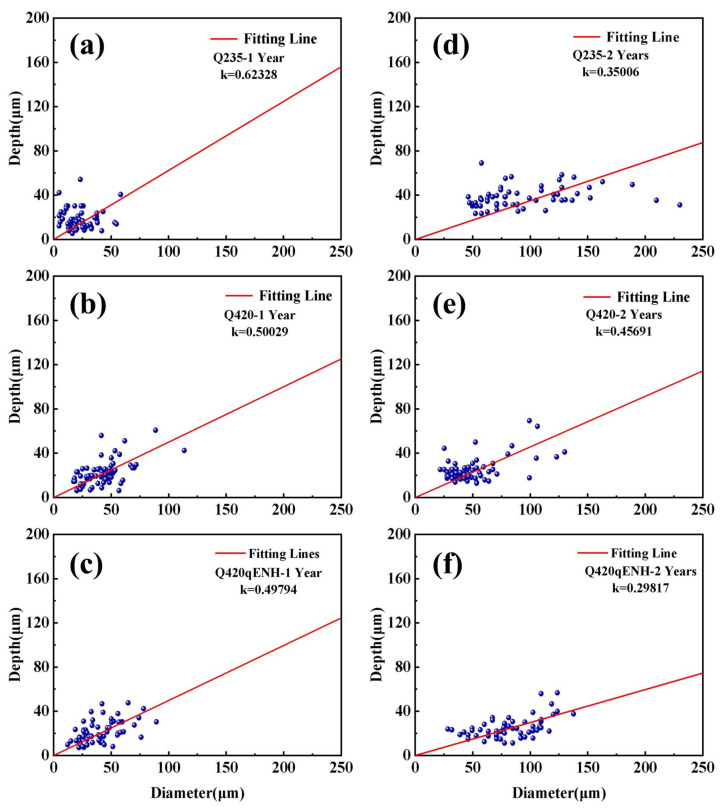
Statistical analysis of pit dimensions (depth vs. diameter) at the arc top regions of descaled U-bend specimens for (**a**–**c**) 1-year and (**d**–**f**) 2-year exposures: (**a**,**d**) Q235, (**b**,**e**) Q420, (**c**,**f**) Q420qENH. Solid lines represent linear fits. The coefficient *k* represents the depth-to-diameter ratio.

For further statistical validation, pit dimensions at the arc top were analyzed by CLSM (Figure 11). After one year, Q235 showed relatively small pits but the highest depth-to-diameter ratio. Q420 and Q420qENH exhibited moderate pit sizes, although pits on Q420qENH were notably shallower. With extended exposure to two years, pit width and depth increased for all steels, while the depth-to-diameter ratios decreased—reflecting a slowdown in vertical growth and a shift toward lateral expansion. Among the three steels, Q235 displayed the most pronounced increase in overall pit size but the largest reduction in depth-to-diameter ratio. Although pits on Q420 remained relatively small, their depth-to-diameter ratio declined the least, pointing to the strongest ongoing tendency for depth-oriented growth. Q420qENH exhibited moderate pit dimensions but a markedly lower depth-to-diameter ratio than both Q235 and Q420, demonstrating superior corrosion resistance under stress. These statistical trends align with the data in Figure 10, confirming the reliability of the derived pitting corrosion behavior.

### 3.4. Cross-Sectional Analysis of Rust Layers

**Figure 12:**
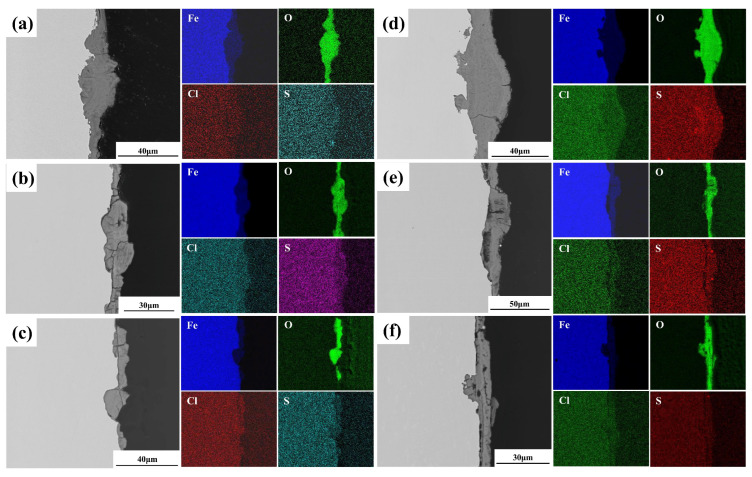
Cross-sectional SEM images and corresponding EDS elemental maps of rust layers on plate specimens after (**a**–**c**) 1-year and (**d**–**f**) 2-year exposures: (**a**,**d**) Q235, (**b**,**e**) Q420, (**c**,**f**) Q420qENH.

Figure 12 presents cross-sectional SEM images and corresponding EDS elemental maps of the rust layers formed on the plate specimens. The rust layer thickness increased with exposure time for both Q235 and Q420 steels. In contrast, while the rust layer on Q420qENH showed no significant thickness increase, it became markedly more uniform and continuous. Oxygen enrichment is evident throughout the rust layers of all steels, confirming that iron oxides/hydroxides constitute the primary corrosion products. Notably, sulfur (S) enrichment was detected within the rust layer at the bottom of pits on Q235 steel after two years of exposure, implying the potential involvement of S in localized corrosion processes.

**Figure 13:**
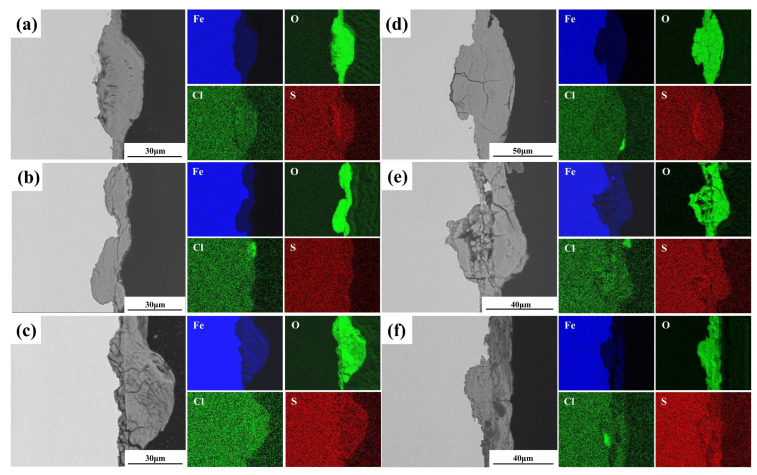
Cross-sectional SEM images and corresponding EDS elemental maps of rust layers at the arc top regions of U-bend specimens after (**a**–**c**) 1-year and (**d**–**f**) 2-year exposures: (**a**,**d**) Q235, (**b**,**e**) Q420, (**c**,**f**) Q420qENH.

Figure 13 presents cross-sectional SEM images and corresponding EDS elemental maps of the rust layers on the U-bend specimens. With prolonged exposure, the rust layer thickness increased noticeably for Q235 and Q420, while remaining largely unchanged for Q420qENH. As expected, significant oxygen enrichment is observed throughout the rust layers of all specimens, consistent with the trend noted for plate specimens (Figure 12). Notably, S enrichment is evident at the rust-layer/substrate interface of the Q235 U-bend specimens after both exposure periods (Figure 13a,d), indicating the involvement of sulfur species in the corrosion process. Furthermore, Cl enrichment is detected in the rust layers of all specimens after two-year exposure (Figure 13d–f), primarily within the outer to middle regions. This distribution suggests that the corrosion products may act as a partial barrier, impeding the inward migration of chloride ions toward the steel substrate and thereby mitigating corrosion to some extent.

### 3.5. Composition Analysis of Rust Layer

**Figure 14:**
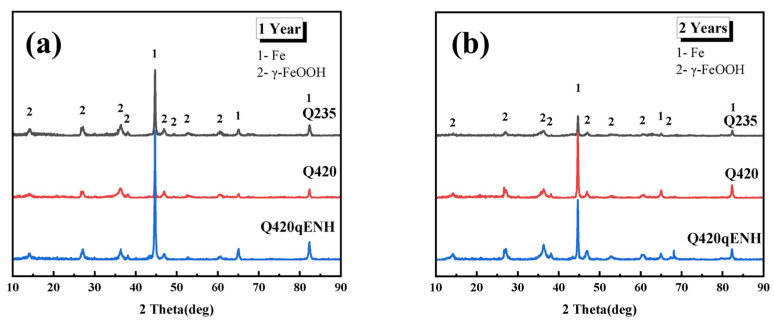
XRD analysis results of the rust layers on plate specimens after (**a**) 1-year and (**b**) 2-year exposures.

The phase composition of corrosion products formed on the plate specimens was analyzed by XRD, with the corresponding patterns shown in Figure 14. The XRD results indicate that γ-FeOOH is the predominant crystalline phase in the rust layers of all three steels. γ-FeOOH is known to form relatively dense, protective layers during long-term atmospheric exposure [29], which aligns with the dense, layered morphologies observed by SEM (Figure 5 and Figure 8). This phase-related densification provides a mechanistic explanation for the decrease in corrosion rate over time for both coupon and U-bend specimens (Figure 4).

### 3.6. Electrochemical Test Results

**Figure 15:**
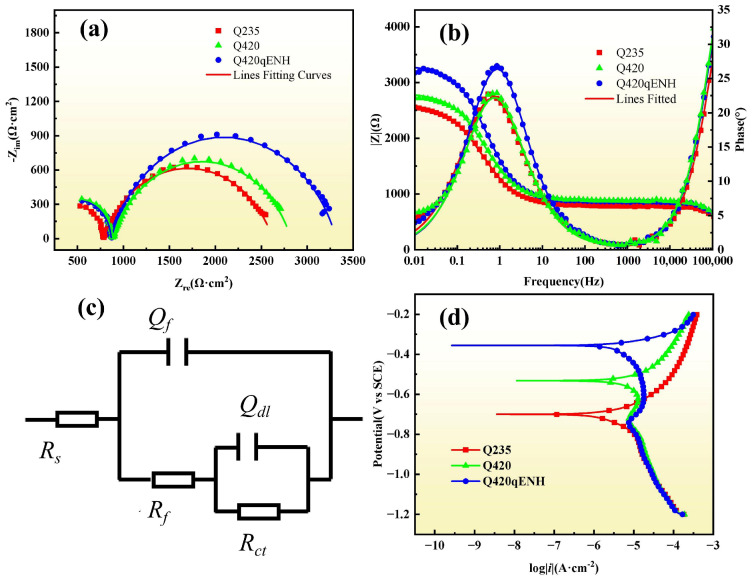
Electrochemical test results in simulated Jinsha River water: (**a**) Nyquist plots, (**b**) Bode phase plots, (**c**) equivalent circuit model for EIS fitting, and (**d**) potentiodynamic polarization curves.

EIS and potentiodynamic polarization measurements of the three steels in simulated Jinsha River water are presented in Figure 15. The Nyquist plots (Figure 15a) show that Q420qENH exhibits the largest impedance arc, reflecting its highest charge-transfer resistance. This result is consistent with the lower corrosion rates and more favorable pit morphology (i.e., lower depth-to-diameter ratio) observed for Q420qENH in field-exposure tests. The Bode phase plots (Figure 15b) reveal two time constants, evidenced by distinct peaks in the high- and low-frequency regions. Accordingly, the EIS data were fitted using the equivalent electrical circuit depicted in Figure 15c, which includes *R_s_* (solution resistance), a parallel *Q_f_* − *R_f_* pair (constant-phase element and resistance of the rust layer), and a parallel *Q_dl_* − *R_ct_* pair (double-layer CPE and charge-transfer resistance). The fitted parameters are summarized in Table 3. The total polarization resistance (*R_f_* + *R_ct_*) is highest for Q420qENH, confirming its superior corrosion resistance in this environment. The reliability of the fitting is supported by *χ^2^* values on the order of 10^−4^.

The potentiodynamic polarization curves (Figure 15d) display similar anodic branches for all steels, characteristic of activation-controlled dissolution. The cathodic branches are governed mainly by the oxygen-reduction reaction; however, those of Q420 and Q420qENH show diffusion-limited behavior, in line with previous reports [31]. The corrosion potentials follow the order *E_corr_*(Q235) < *E_corr_*(Q420) < *E_corr_*(Q420qENH), indicating that Q420qENH possesses the lowest thermodynamic tendency to corrode in this environment—further corroborating its enhanced corrosion resistance.

## 4. Discussion

### 4.1. Analysis of Corrosion Mechanisms in Structural Steel Under Plateau Valley Atmospheric Conditions

Consistent with previous studies [29,32,33] and the polarization curves measured in this work (Figure 15d), the atmospheric corrosion of iron proceeds through the formation of hydrated oxides. The overall process involves the anodic dissolution of iron coupled with the predominantly cathodic reduction of oxygen, represented by the following half-cell reactions [29]:

(3) $$Fe\to Fe^{2+}+2e^{-}$$

(4) $$O_{2}+4e^{-}+2H_{2}O\to 4OH^{-}$$

These two half-cell reactions result in the formation of Fe(OH)_2_ [29]:

(5) $$Fe^{2+}+2e^{-}+4OH^{-}\to 2Fe{\left(OH\right)}_{2}$$

As corrosion time increases, Fe(OH)_2_ undergoes further oxidation in the atmospheric environment, forming the metastable γ-FeOOH [29]:

(6) $$4Fe{\left(OH\right)}_{2}+O_{2}\to 4\gamma -FeOOH+2H_{2}O$$

γ-FeOOH may further transform into thermodynamically stable goethite (α-FeOOH) via solid-state phase transitions, or form magnetite (Fe_3_O_4_) under oxygen-deficient conditions [29]:

(7) γ−FeOOH→α−FeOOH

(8) $$8\gamma -FeOOH+Fe\to 3Fe_{3}O_{4}+4H_{2}O$$

In this study, γ-FeOOH was identified as the primary crystalline corrosion product on all three steels exposed to the plateau valley atmosphere (Figure 12, Figure 13 and Figure 14). Although the rust layer coverage and thickness increased over time, no α-FeOOH or Fe_3_O_4_ was detected. This indicates that, in contrast to some previous studies, the phase-transformation kinetics in this specific plateau valley environment are significantly slower, impeding the conversion of γ-FeOOH to α-FeOOH and Fe_3_O_4_ described by Equations (7) and (8). The relatively low temperature and the low chloride deposition rate characteristic of this plateau valley environment may slow the phase transformation kinetics of the corrosion products, thereby inhibiting the conversion of γ-FeOOH to more stable phases such as α-FeOOH or Fe_3_O_4_. Consequently, these phases remained undetectable even after two years of exposure.

The protective role of γ-FeOOH rust layers in plateau valley atmospheres remains unclear. In the present work, although the uniform corrosion rate decreased over time (Figure 4), the depth-to-diameter ratio of pits slightly increased with prolonged exposure (Figure 7), indicating continued propagation of localized corrosion in the depth direction. This divergence suggests that while the surface γ-FeOOH layer offers some general protection, the microenvironment within pits becomes oxygen-depleted and locally acidified [34], favoring sustained pit deepening. Thus, in the plateau valley environment, the γ-FeOOH layer provides only limited protection: it slows uniform corrosion but is less effective at suppressing the deepening of existing pits.

Sulfur enrichment was observed in the rust layer of Q235 specimens (Figure 12d and Figure 13a,d), pointing to the involvement of sulfur in the corrosion process. The atmospheric SO_2_ concentration in Baiyu County is approximately 6 μg/m^3^ according to the historical records provided by the meteorological bureau, indicating a relatively low but measurable level of SO_2_ in the local atmosphere. The sulfur likely originates from atmospheric SO_2_, which dissolves in the thin surface electrolyte film under humid conditions to form sulfite ions (SO_3_^2−^) [35]. These can be further oxidized to sulfate ions (SO_4_^2−^) [14], which subsequently react with anodically generated Fe^2+^ to form ferrous sulfate (FeSO_4_) [36,37]:

(9) $$2SO_{3}^{2-}+O_{2}\to 2SO_{4}^{2-}$$

(10) $$SO_{4}^{2-}+Fe^{2+}\to FeSO_{4}$$

FeSO_4_ can generate the following cyclic corrosion process in the atmosphere [37]:

(11) $$4FeSO_{4}+O_{2}+6H_{2}O\to 4FeOOH+4H_{2}SO_{4}$$

(12) $$2H_{2}SO_{4}+O_{2}+2Fe\to 2FeSO_{4}+2H_{2}O$$

The resulting H_2_SO_4_ can permeate the rust layer, establishing ion-conducting pathways that promote the inward transport of oxygen and other aggressive species. This mechanism sustains both anodic dissolution (Reaction (3)) and cathodic oxygen reduction (Reaction (4)) at the steel interface, thereby accelerating the overall corrosion process.

Furthermore, chlorine enrichment was observed in the rust layers of all U-bend specimens after two years of exposure (Figure 13d–f), which may attributed to the deposition of atmospheric chloride-containing species. Cl^−^ that reach the steel substrate may engage in the following detrimental reactions [5,38]:

(13) $$Fe^{2+}+Cl^{-}+OH^{-}\to FeOCl+HCl$$

In atmospheric environments, structural steel frequently experiences alternating dry and wet cycles, which will lead to [38]:

(14) FeOCl→β−FeOOH

Akaganéite (β-FeOOH) is known to degrade the protectiveness of rust layers [39], as it can sustain the inward transport of aggressive species to the steel substrate, thereby perpetuating corrosion reactions (e.g., Equations (4)–(7)). It is known that β-FeOOH typically forms in chloride-rich environments. Although the exact threshold for akaganeite formation varies with exposure conditions, the absence of detectable β-FeOOH in all specimens (Figure 14) suggests that the local chloride concentration at the study site was below the critical level required for its formation. This is consistent with the inland location of the exposure site and the lack of direct marine influence. Cross-sectional EDS maps (Figure 13d–f), however, reveal a distinct distribution of Cl: it is enriched mainly on the outer rust-layer surface in Q235 and Q420 steels, but within the middle layer in Q420qENH. This distribution indicates that the rust layers on all three steels act as a partial barrier to chloride ingress. By impeding Cl^−^ from reaching the steel–rust interface, this barrier effect likely suppresses the formation of detrimental β-FeOOH.

### 4.2. Effect of Stress on the Corrosion Behavior of Structural Steel in the Plateau Valley Atmospheric Environment

Under the combined action of corrosive media and applied tensile stress, stress concentrations develop at the bottom of surface pits, inducing localized plastic deformation. This deformation disrupts the formation of a stable, protective corrosion product layer, thereby maintaining a fresh and electrochemically active metal surface exposed to the corrosive environment. Consequently, the anodic dissolution rate (Reaction (4)) is enhanced, accelerating the overall corrosion process [40]. Moreover, sustained stress concentration can trigger microcrack initiation, whose unstable propagation may lead to accelerated substrate failure [41,42].

A direct comparison of corrosion rates between plate and U-bend specimens (Figure 4) clearly demonstrates that applied stress significantly accelerates corrosion, with U-bend specimens exhibiting markedly higher rates—consistent with prior reports on stress-accelerated atmospheric corrosion [43]. Pits formed at the arc top regions of U-bend specimens (Figure 10 and Figure 11) are considerably deeper and possess higher depth-to-diameter ratios than those on plate specimens (Figure 7). This indicates that applied stress hinders the accumulation of protective corrosion products at the pit bottom, reducing the rust layer’s effectiveness and promoting pit deepening. Such deepened pits act as more severe stress concentrators and preferred sites for microcrack nucleation, thereby increasing the threat to structural integrity [30]. Notably, the depth-to-diameter ratios of pits at the U-bend arc top decrease with prolonged exposure (Figure 9d–f), a trend opposite to that observed for coupons. This reversal may be attributed to tensile stress disrupting the protective corrosion-product layer on the pit walls, which weakens local protection and shifts corrosion attack laterally, leading to pit widening.

Furthermore, sulfur enrichment was detected in the rust layer of Q235 plate specimens only after two years (Figure 12d), but was already evident in Q235 U-bend specimens after just one year (Figure 13d). This accelerated sulfur ingress suggests that tensile stress enhances the transport of sulfur species through the rust layer, facilitating their earlier involvement in corrosion-accelerating reactions (e.g., Equations (9)–(13)).

### 4.3. Differences in Corrosion Behavior of Various Structural Steels in the Atmospheric Environment of Plateau Valleys

Based on corrosion rate (Figure 4) and electrochemical performance (Figure 15), the corrosion resistance of the three steels in the plateau valley atmosphere is ranked as: Q420qENH > Q420 > Q235.

The chemical compositions (Table 1) reveal distinct alloying strategies: compared to the plain carbon steel Q235, Q420 contains minor additions of Mo, Ni, and Cr, while Q420qENH is alloyed with Cu and higher levels of Ni and Cr. Alloying with Ni and Mo is known to promote the formation of protective NiFe_2_O_4_ spinel in the rust layer, which can catalyze the transformation to more stable phases (e.g., via Equation (7)) and enhance protectiveness, thereby reducing corrosion rates [15,19,21]. However, XRD analysis (Figure 14) detected only γ-FeOOH, with no NiFe_2_O_4_ identified. This suggests that under the specific conditions of the plateau valley atmosphere, the ability of Ni and Mo to promote protective spinel formation is significantly attenuated. Consequently, the superior corrosion resistance of Q420qENH cannot be primarily attributed to Ni and Mo addition in this environment.

Instead, copper (Cu) addition plays a more critical role. As demonstrated by Wang et al. [44], Cu enhances the densification and interfacial stability of the corrosion product layer, effectively blocking chloride ingress and mitigating intergranular attack. This mechanism is corroborated by our experimental observations: the rust layer on Q420qENH is noticeably denser (Figure 5 and Figure 8), and its pits exhibit lower depth-to-diameter ratios (Figure 7 and Figure 11), both indicating a more protective rust layer. Hence, the superior corrosion resistance of Q420qENH is predominantly attributed to its copper alloying content.

Beyond composition, microstructure significantly influences the corrosion behavior [45,46,47,48]. Q235 and Q420 exhibit a ferrite–pearlite microstructure, while Q420qENH features a bainitic structure (Figure 3). Coarse pearlite, as in Q235, is susceptible to preferential dissolution [46]. This localized attack can generate internal stresses within the overlying rust layer, promoting its cracking and delamination [47], thereby compromising corrosion resistance. This microstructurally induced rust layer instability may further explain the more pronounced sulfur enrichment observed in stressed Q235 specimens (Section 4.2), as cracks and defects provide pathways for sulfur ingress. In Q420, the refined ferrite–pearlite structure mitigates pronounced pearlite dissolution, resulting in lower internal stress in the rust layer and consequently better protection than Q235. For Q420qENH, the bainitic structure promotes the formation of a denser, more uniform, and adherent corrosion product layer [47,48], offering the highest degree of substrate protection and hence the best overall corrosion resistance.

Under applied stress, the mechanical strength of the steels introduces another factor influencing corrosion behavior. The mechanical strength of the three steels is reflected in their grade designations: Q235 has a nominal yield strength of approximately 235 MPa, while both Q420 and Q420qENH have a nominal yield strength of approximately 420 MPa, indicating a substantially higher-strength level than Q235. Higher-strength materials generally show increased susceptibility to corrosion-assisted cracking mechanisms, such as corrosion fatigue and SCC, which is often linked to a higher density of microstructural defects introduced during processing [49,50,51]. Consistent with this, the U-bend specimens of Q420 steel exhibited a stronger tendency for deep pit growth compared to Q235 (Figure 10 and Figure 11), indicating a higher propensity for stress corrosion cracking (SCC). Notably, despite having a similar strength level to Q420, Q420qENH exhibited the lowest pit depth-to-diameter ratio. This superior performance underscores the dominant role of its corrosion-resistant design (via Cu alloying and bainitic microstructure), which fosters a denser rust layer that effectively suppresses pit propagation in all directions, outweighing the intrinsic strength-related susceptibility.

In summary, the atmospheric corrosion of the three structural steels in the plateau valley atmospheric environment follows a similar electrochemical mechanism but yields markedly different performance outcomes. O_2_ serves as the main cathodic depolarizer, with sulfur dioxide SO_2_ also contributing to the corrosion process, especially by promoting localized corrosion. Applied tensile stress synergistically accelerated degradation, evidenced by increased uniform corrosion rates, elevated pit depth-to-diameter ratios (reflecting a shift toward depth-wise growth), and a heightened tendency for micro-crack initiation. Corrosion resistance and pitting corrosion behavior are controlled by a combination of alloy composition, microstructure, and material strength, where corrosion-resistant design effectively counterbalances the detrimental effect of higher strength.

This work systematically examines the atmospheric corrosion behavior of typical engineering structural steels in the plateau valley atmospheric environment. The elucidated corrosion mechanisms, performance rankings, and quantitative data establish a critical foundation for material selection, durability prediction, and anti-corrosion design of infrastructure in similar environments.

## 5. Conclusions

This study systematically investigated the corrosion behavior and mechanisms of three typical structural steels (Q235, Q420, Q420qENH) in a plateau valley atmospheric through field exposure tests (using plate and U-bend specimens), electrochemical analysis, and characterization. The principal conclusions are as follows:

1. Uniform corrosion was the primary corrosion mode of the three steels, with pitting corrosion as the secondary form, regardless of the presence of stress. Q420qENH exhibited the lowest corrosion rate and the best overall resistance, outperforming Q235 and Q420. Its superior performance is attributed primarily to copper alloying, which promotes a denser, more homogeneous, and protective rust layer.
2. Applied tensile stress significantly accelerated both uniform and localized corrosion. Compared with stress-free plate specimens, the U-bend specimens showed higher uniform corrosion rates, greater maximum pit depths, and elevated pit depth-to-diameter ratios. The stress-induced acceleration of localized corrosion was most pronounced for Q420 steel.
3. In this atmosphere, the rust layer acted as a partial barrier, retarding the ingress of Cl^−^ toward the steel substrate. However, interaction with SO_2_ created diffusion pathways for aggressive species within the rust layer, exacerbating its breakdown at pit bottoms and causing local acidification, thereby further degrading corrosion resistance.

## Figures and Tables

**Table 1:** Chemical composition of the three structural steels (wt.%).

Element	C	Mn	Si	P	S	Ni	Cr	Cu	Mo	Fe
Q235	0.085	1.523	0.180	0.010	0.003	0.024	0.021	0.030	0.010	Bal.
Q420	0.071	1.481	0.333	0.008	0.004	0.147	0.228	0.019	0.164	Bal.
Q420qENH	0.082	1.139	0.218	0.013	0.002	0.348	0.540	0.362	0.092	Bal.

**Table 2:** Main chemical composition of the simulated Jinsha River water environment (mg/L).

Composition	MgCl_2_·6H_2_O	CaCl_2_	Na_2_CO_3_	NaHCO_3_	KCl	Na_2_SO_4_
Concentration(mg/L)	20.77	113.73	8.83	182.84	1.88	21.32

**Table 3:** Equivalent circuit fitting parameters obtained from EIS tests.

Parameters	R_s_ (Ω·cm^2^)	R_f_ (Ω·cm^2^)	Q_f_·10^−4^ (Ω^−1^cm^−2^s^n^)	n_f_	Q_dl_·10^−4^ (Ω^−1^cm^−2^s^n^)	n_dl_	R_ct_ (Ω·cm^2^)	χ^2^· 10^−4^
Q235	162.5	621.8	3.39 × 10^−5^	0.8	3.33	0.8	1840	1.20
Q420	157.1	726.2	2.86 × 10^−9^	0.9698	2.76	0.7735	1940	2.90
Q420qENH	164.3	701.4	2.95 × 10^−9^	0.9694	2.05	0.7989	2448	1.57

## Data Availability

The original contributions presented in this study are included in the article. Further inquiries can be directed to the corresponding author.
